# A 3D-Printed BCP/PLGA-PEG-PLGA Scaffold for LNC000133-Loaded BMSC-Derived Exosome Delivery Promotes Repair of Steroid-Induced Osteonecrosis of the Femoral Head

**DOI:** 10.3390/ijms27209277

**Published:** 2026-10-19

**Authors:** Tixiong Xia, Chengbin Yang, Xi Li, Tong Chen, Yingxing Xu

**Affiliations:** 1 Department of Orthopaedics, First Affiliated Hospital of Kunming Medical University, Kunming 650032, China; 20240543@kmmu.edu.cn (T.X.); 20220450@kmmu.edu.cn (C.Y.); lixi@ydyy.cn (X.L.); 2 Research Center for Clinical Medicine, First Affiliated Hospital of Kunming Medical University, Kunming 650032, China; chentong1@kmmu.edu.cn

**Keywords:** biphasic calcium phosphate scaffold, Exosomes, lncRNA LNC000133, osteogenesis, angiogenesis, steroid-induced osteonecrosis of the femoral head

## Abstract

Dysfunction of bone marrow mesenchymal stem cells (BMSCs) and impaired exosome-mediated intercellular communication are recognized as critical contributors to the pathogenesis of steroid-induced osteonecrosis of the femoral head (SONFH), although the underlying mechanisms remain incompletely understood. Moreover, the development of novel biomaterials and therapeutic strategies capable of improving SONFH treatment remains an urgent clinical priority. Previous studies have demonstrated that LNC000133-modified exosomes derived from human BMSCs (hBMSCs) promote osteoblastic differentiation and enhance exosome uptake by osteoblasts, thereby exerting therapeutic effects in SONFH. Building on these findings, we developed a 3D-printed biphasic calcium phosphate (BCP)/poly (lactic-co-glycolic acid) (PLGA)- polyethylene glycol (PEG)-PLGA scaffold loaded with LNC000133-modified hBMSC-derived exosomes (LNC000133-Exos) to facilitate the clinical translation of this gene-based therapeutic approach for SONFH. The biological effects of this composite scaffold on hBMSCs were subsequently evaluated in vitro. Furthermore, the BCP/PLGA-PEG-PLGA scaffold loaded with LNC000133-Exos was applied for the treatment of SONFH in a rabbit model. Our results demonstrated that transplantation of the LNC000133-Exos-loaded BCP/PLGA-PEG-PLGA scaffold markedly enhanced bone repair and structural reconstruction within the necrotic region of the femoral head in vivo. Collectively, these findings indicate that the BCP/PLGA-PEG-PLGA scaffold and LNC000133 synergistically promote hBMSC proliferation and osteogenic differentiation while suppressing adipogenic differentiation, providing a strong theoretical foundation and a promising therapeutic strategy for targeted SONFH treatment.

## 1. Introduction

Steroid-induced osteonecrosis of the femoral head (SONFH), a major form of non-traumatic osteonecrosis associated with excessive glucocorticoid exposure, is characterized by increasing incidence, substantial disability, and a high prevalence of bilateral involvement [1,2]. The pathogenesis of SONFH is multifactorial and remains incompletely understood, involving impaired bone remodeling, microvascular dysfunction, and dysregulated lipid metabolism resulting from compromised blood supply to the femoral head [3,4]. These pathological changes collectively drive disease progression and pose significant challenges to hip-preserving therapeutic strategies for SONFH. In this context, exosomes derived from bone marrow mesenchymal stem cells (BMSC-derived exosomes, BMSC-Exos) have attracted considerable attention as potential cell-free therapeutic mediators because of their excellent biocompatibility, natural cargo-delivery capacity, and ability to facilitate intercellular communication. Increasing evidence supports their potential roles in tissue repair and bone regeneration [5,6,7,8].

In our previous study, we comprehensively characterized the differential expression profiles of lncRNAs, miRNAs, and mRNAs in exosomes derived from human BMSCs (hBMSCs) associated with SONFH and identified the novel lncRNA LNC000133 as a potential regulatory molecule. Furthermore, our previous study demonstrated that LNC000133-modified hBMSC-derived exosomes enhanced osteoblast proliferation and osteogenic differentiation through the miR-362-5p/TGF-β3/SMAD3/BMP2 signaling axis [9]. These findings support the therapeutic potential of LNC000133-modified exosomes for promoting bone regeneration. However, effective delivery and sustained retention of exosomes at the target site remain important challenges for their application to localized skeletal lesions. Systemic administration, particularly intravenous delivery, has been widely explored in exosome-based therapies [10,11], but may be less suitable for a localized lesion such as SONFH because maintaining sufficient exosome exposure within the necrotic femoral head remains challenging. Core decompression, a well-established hip-preserving procedure, not only reduces intraosseous pressure and improves local blood perfusion but also provides an anatomical route for local delivery to the affected femoral head [12,13]. Therefore, a delivery system capable of retaining exosomes within the core decompression tunnel and maintaining their local availability is of particular interest for SONFH treatment.

Biomaterial-based delivery systems, including hydrogels and scaffolds, have been increasingly explored to provide localized and sustained delivery of therapeutic agents. In our previous study, a poly(lactic-co-glycolic acid) (PLGA)-polyethylene glycol (PEG)-PLGA hydrogel encapsulating BMSCs was implanted into the core decompression tunnel, establishing a localized sustained-release system that achieved promising therapeutic outcomes [2]. More recently, injectable thermosensitive PLGA-PEG-PLGA hydrogels combined with core decompression have also been investigated for sustained local delivery in SONFH [12]. Nevertheless, hydrogel-based systems alone provide limited mechanical support for the necrotic femoral head. Biphasic calcium phosphate (BCP) scaffolds provide structural support and osteoconductive properties and have been investigated as biomaterial platforms for bone regeneration and local delivery of bioactive agents [14,15,16,17]. Leveraging the thermoresponsive properties of PLGA-PEG-PLGA hydrogels [18] together with the structural and osteoconductive properties of BCP scaffolds, we developed a composite BCP/PLGA-PEG-PLGA scaffold for the local delivery of LNC000133-modified hBMSC-derived exosomes. In the present study, we investigated this composite system as an adjunct to core decompression for SONFH and evaluated its biological effects and regenerative potential in vitro and in vivo.

## 2. Results

### 2.1. Physicochemical Characterization of the BCP/PLGA-PEG-PLGA Scaffold for Implantation

**Figure 1:**
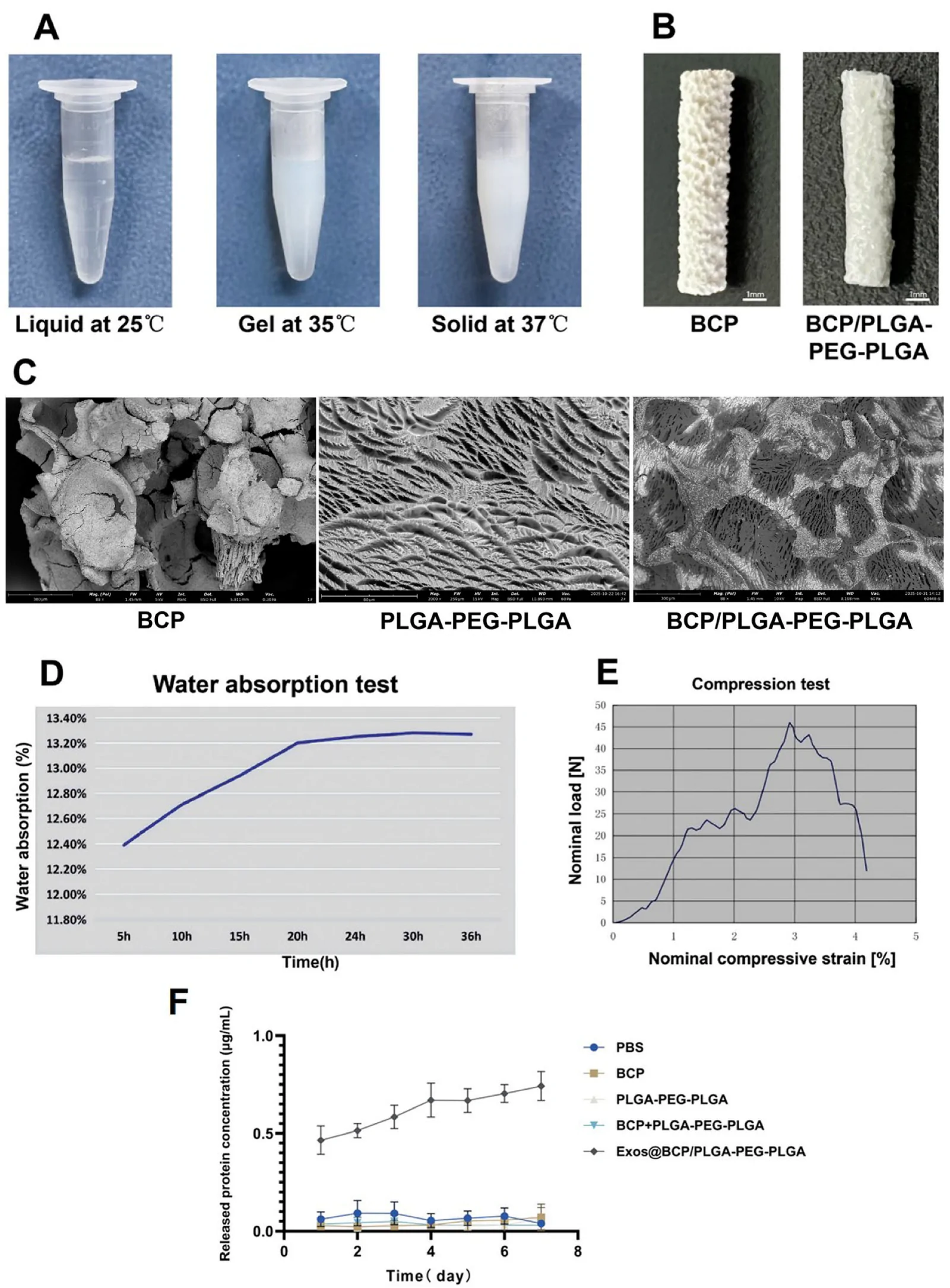
**Physicochemical characterization of the BCP/PLGA-PEG-PLGA scaffold system.** (**A**) Thermoresponsive phase transition of PLGA-PEG-PLGA hydrogel at different temperatures; (**B**) morphological comparison of pristine BCP scaffolds and PLGA-PEG-PLGA hydrogel-infiltrated BCP scaffolds (scale bar = 1 mm); (**C**) scanning electron microscopy images of the BCP scaffold; (**D**) Water absorption profile of the scaffold over time; (**E**) compression load–strain curve of the scaffold. (**F**) Released protein concentrations of different scaffold formulations over 7 days. Note: BCP, biphasic calcium phosphate; PLGA-PEG-PLGA, poly (lactic-co-glycolic acid)-polyethylene glycol–poly (lactic-co-glycolic acid).

As shown in Figure 1A, the PLGA-PEG-PLGA hydrogel remained free-flowing at 25 °C, gradually transformed into a semi-transparent gel with decreased fluidity at 35 °C, and became a white, non-flowing solid at temperatures ≥37 °C. As shown in Figure 1B, the pristine 3D-printed BCP scaffold exhibited a highly porous architecture with well-interconnected pores, whereas the PLGA-PEG-PLGA hydrogel-infiltrated BCP scaffold displayed partial pore filling and a uniform surface coating while preserving the overall scaffold integrity. The scaffold exhibited a porosity of 65 ± 10%, with a diameter of 3 mm and a length of 10 ± 0.5 mm. The central through-pore had a diameter of 1 ± 0.5 mm. SEM further revealed that the BCP scaffold consisted of irregularly assembled particles with interconnected pores and a rough surface containing micro- and submicron-scale structures, providing a suitable platform for exosome adsorption and distribution within the scaffold (Figure 1C). As shown in Figure 1D, water absorption by the BCP scaffold increased over time and reached a stable plateau of 13.2% at approximately 30 h. Furthermore, the BCP scaffold exhibited an approximately linear elastic response at low strains, followed by a progressive increase in load until reaching a maximum value of 45–46 N at 3% strain. A subsequent rapid decline in load indicated structural failure of the scaffold (Figure 1E). To further evaluate the release behavior of the scaffold system, the protein concentration in the release medium was measured by the BCA assay over 7 days. The LNC000133-Exos@BCP/PLGA-PEG-PLGA scaffolds showed a gradual increase in released protein concentration during the first 4 days, followed by a relatively stable level thereafter (Figure 1F). The initial increase may reflect progressive hydration of the PLGA-PEG-PLGA hydrogel and diffusion of exosome-associated proteins through the hydrogel network and porous BCP structure. The subsequent plateau suggested a relatively stable release phase, supporting the sustained release capability of the composite scaffold. In contrast, the PBS, BCP, PLGA-PEG-PLGA, and BCP/PLGA-PEG-PLGA groups exhibited only low-level protein release throughout the observation period.

### 2.2. Identification of hBMSCs

**Figure 2:**
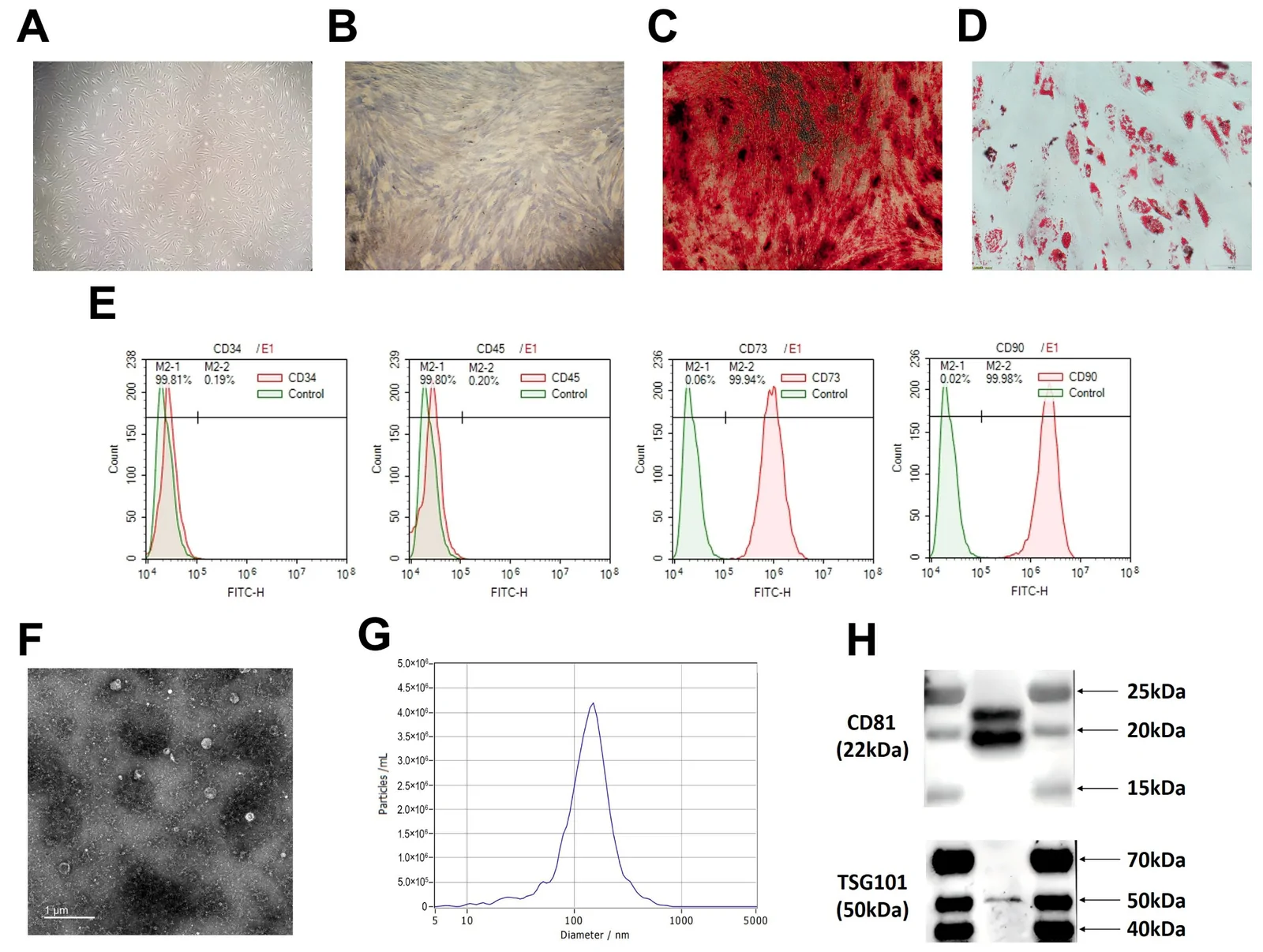
**Characterization of hBMSCs and hBMSC-derived exosomes.** (**A**) Representative images showing the morphology of hBMSCs under an inverted phase-contrast microscope (scale bar, 500 μm); (**B**) ALP staining (scale bar, 500 μm); (**C**) ARS staining (scale bar, 500 μm); (**D**) ORO staining (scale bar, 100 μm); (**E**) flow cytometric analysis of hBMSC phenotypes; (**F**) TEM images of hBMSC-derived exosomes; (**G**) size distribution and concentration profile of exosomes determined by NTA. The *x*-axis represents particle diameter (nm), and the *y*-axis represents particle concentration (particles/mL). The peak indicates the most abundant particle size, whereas the peak width reflects the uniformity of particle size distribution. (**H**) Western blot analysis of the exosome-associated markers TSG101 and CD81 in hBMSC-derived exosomes. Note: hBMSCs, human bone marrow mesenchymal stem cells; hBMSC-derived exosomes, exosomes derived from human bone marrow mesenchymal stem cells; ALP, alkaline phosphatase; ARS, Alizarin Red S; ORO, Oil Red O; TEM, transmission electron microscopy; NTA, nanoparticle tracking analysis.

Cells at passages 3–4 exhibited a typical fibroblast-like morphology, characterized by an elongated spindle-shaped appearance, distinct cell boundaries, and strong light refraction. The cells displayed radial and whirlpool-like growth patterns (Figure 2A). Following osteogenic induction, extensive mineralized matrix deposition and significantly increased ALP activity were observed, indicating successful osteogenic differentiation (Figure 2B,C). Under adipogenic induction conditions, intracellular lipid accumulation was evident, as demonstrated by increased lipid droplet formation in both number and size (Figure 2D). Flow cytometric analysis further revealed that the isolated cells exhibited high expression levels of the mesenchymal stem cell markers CD73 (99.94%) and CD90 (99.98%), while showing minimal expression of hematopoietic markers CD34 (0.19%) and CD45 (0.20%), confirming their mesenchymal stem cell phenotype (Figure 2E).

### 2.3. Identification of hBMSC-Derived Exosomes

Exosomes isolated from hBMSC-conditioned medium were characterized by TEM, which revealed the characteristic cup-shaped morphology of exosomes (Figure 2F). NTA demonstrated that the vesicle size distribution was predominantly within the range of 50–200 nm, with a modal diameter of 138.3 nm and a median diameter of 114.7 nm. The particle concentration was 6.1 × 10^11^ particles/mL, consistent with the typical size distribution and abundance reported for exosomes (Figure 2G). Western blotting further detected the exosome-associated markers TSG101 and CD81 in the isolated exosomes (Figure 2H).

### 2.4. Regulatory Effects of the BCP/PLGA-PEG-PLGA Scaffold Loaded with LNC000133-Exos on hBMSC Proliferation and Apoptosis

**Figure 3:**
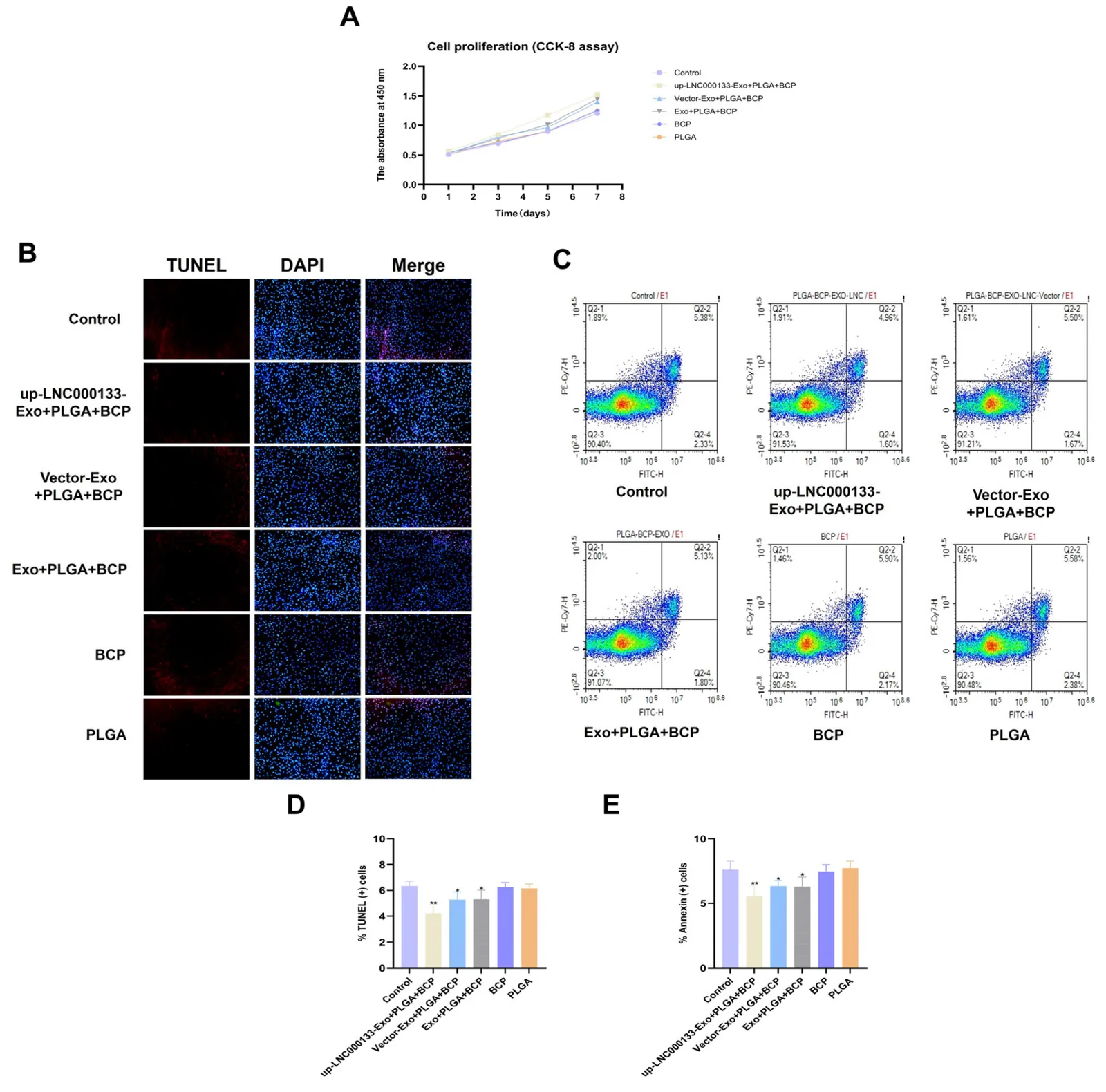
**Effects of BCP/PLGA-PEG-PLGA scaffolds loaded with LNC000133-modified exosomes on cell proliferation and apoptosis.** (**A**) Cell proliferation of hBMSCs assessed using the CCK-8 assay; (**B**) Representative images of TUNEL staining; (**C**) Representative flow cytometric plots showing apoptotic cell populations; (**D**) Quantification of TUNEL-positive cells. (**E**) Quantification of Annexin-positive cells. Data are presented as mean ± standard deviation (SD). Statistical significance was determined by comparison with the Control group: * *p* < 0.05 and ** *p* < 0.01. Abbreviations: LNC000133, long non-coding RNA 000133; PLGA, poly (lactic-co-glycolic acid); BCP, biphasic calcium phosphate.

CCK-8 assays demonstrated significantly enhanced proliferation in the up-LNC000133-Exo+PLGA+BCP group, whereas no significant inhibitory effects on proliferation were observed in the BCP scaffold or PLGA-PEG-PLGA hydrogel groups. Apoptosis assays further revealed a significant reduction in apoptosis in the up-LNC000133-Exo+PLGA+BCP group, whereas neither the BCP scaffold nor the PLGA-PEG-PLGA hydrogel induced significant apoptotic responses. These findings demonstrated that the LNC000133-Exos-loaded BCP/PLGA-PEG-PLGA scaffold promoted hBMSC proliferation and suppressed apoptosis (Figure 3).

### 2.5. Regulatory Effects of the BCP/PLGA-PEG-PLGA Scaffold Loaded with LNC000133-Exos on Osteogenic and Adipogenic Differentiation of hBMSCs

**Figure 4:**
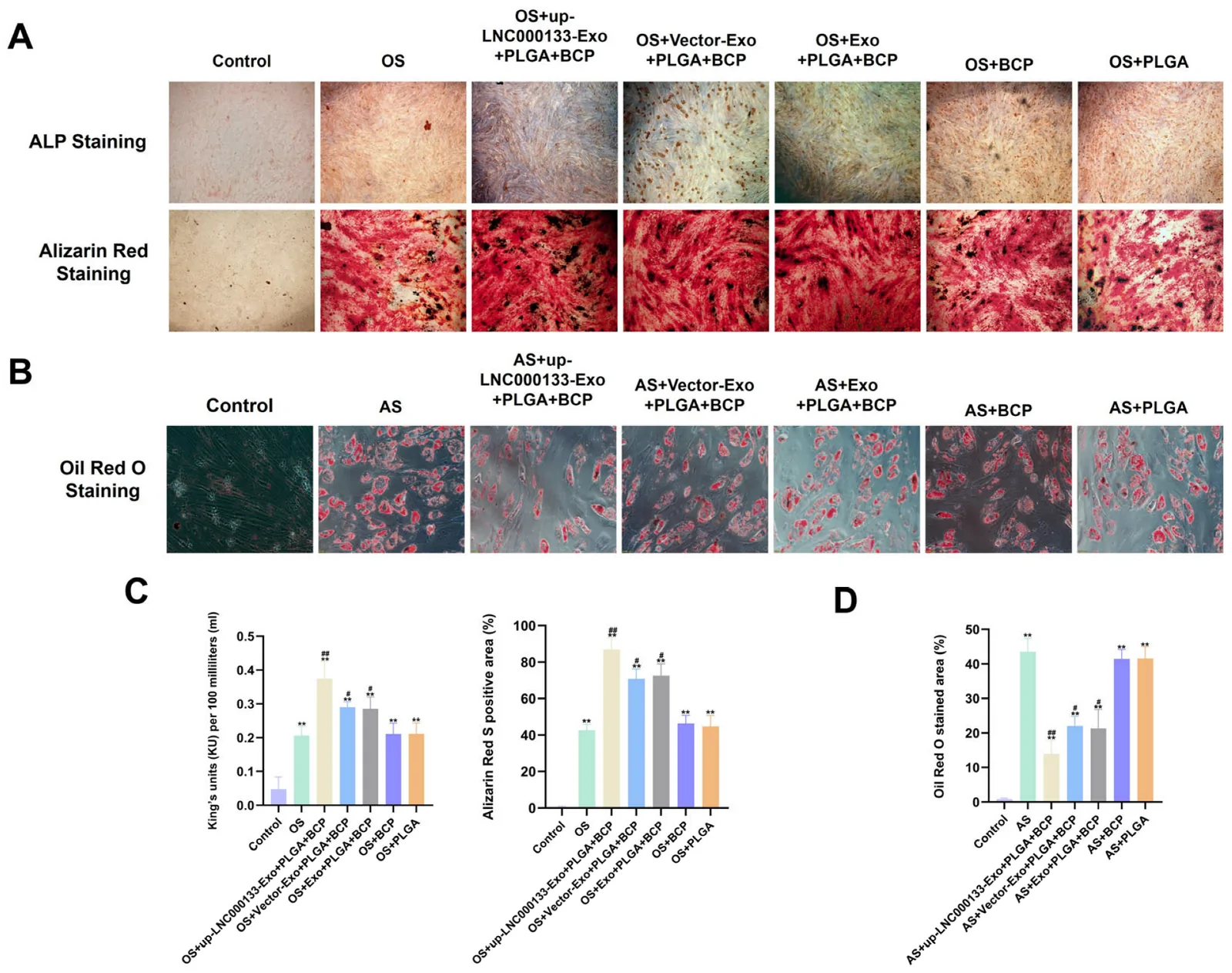
**Effects of LNC000133-Exos-loaded BCP/PLGA-PEG-PLGA scaffolds on osteogenic and adipogenic differentiation.** (**A**) Representative images of osteogenic differentiation, including ALP and ARS staining. (**B**) Representative images of ORO staining. (**C**) Quantitative analysis of osteogenic differentiation. (**D**) Quantitative analysis of lipid accumulation. Data are presented as mean ± standard deviation (SD). Note: * *p* < 0.05 and ** *p* < 0.01 compared with the Control group; ^#^ *p* < 0.05 and ^##^ *p* < 0.01 compared with the OS or AS group. OS, cells were cultured in osteogenic induction medium; AS, cells were cultured in adipogenic induction medium; LNC000133, long non-coding RNA 000133; PLGA, poly (lactic-co-glycolic acid); BCP, biphasic calcium phosphate; ALP, alkaline phosphatase; ARS, Alizarin Red S; ORO, Oil Red O.

ALP staining revealed enhanced ALP activity in the Exo+PLGA+BCP group, whereas weak ALP staining was observed in the control group, consistent with the results of the ALP activity assay. ARS staining demonstrated increased mineralized nodule formation in the Exo+PLGA+BCP group, whereas limited calcium deposition was observed in the control group. ORO staining revealed reduced lipid droplet accumulation in the Exo+PLGA+BCP group, whereas abundant lipid droplets were detected in the control group. These findings demonstrated that the LNC000133-Exos-loaded BCP/PLGA-PEG-PLGA scaffold enhanced osteogenic differentiation, as indicated by increased ALP activity and mineralized nodule formation, while suppressing adipogenic differentiation of hBMSCs (Figure 4).

### 2.6. Imaging Evaluation of LNC000133-Exos-Loaded BCP/PLGA-PEG-PLGA Scaffolds in SONFH Rabbits

**Figure 5:**
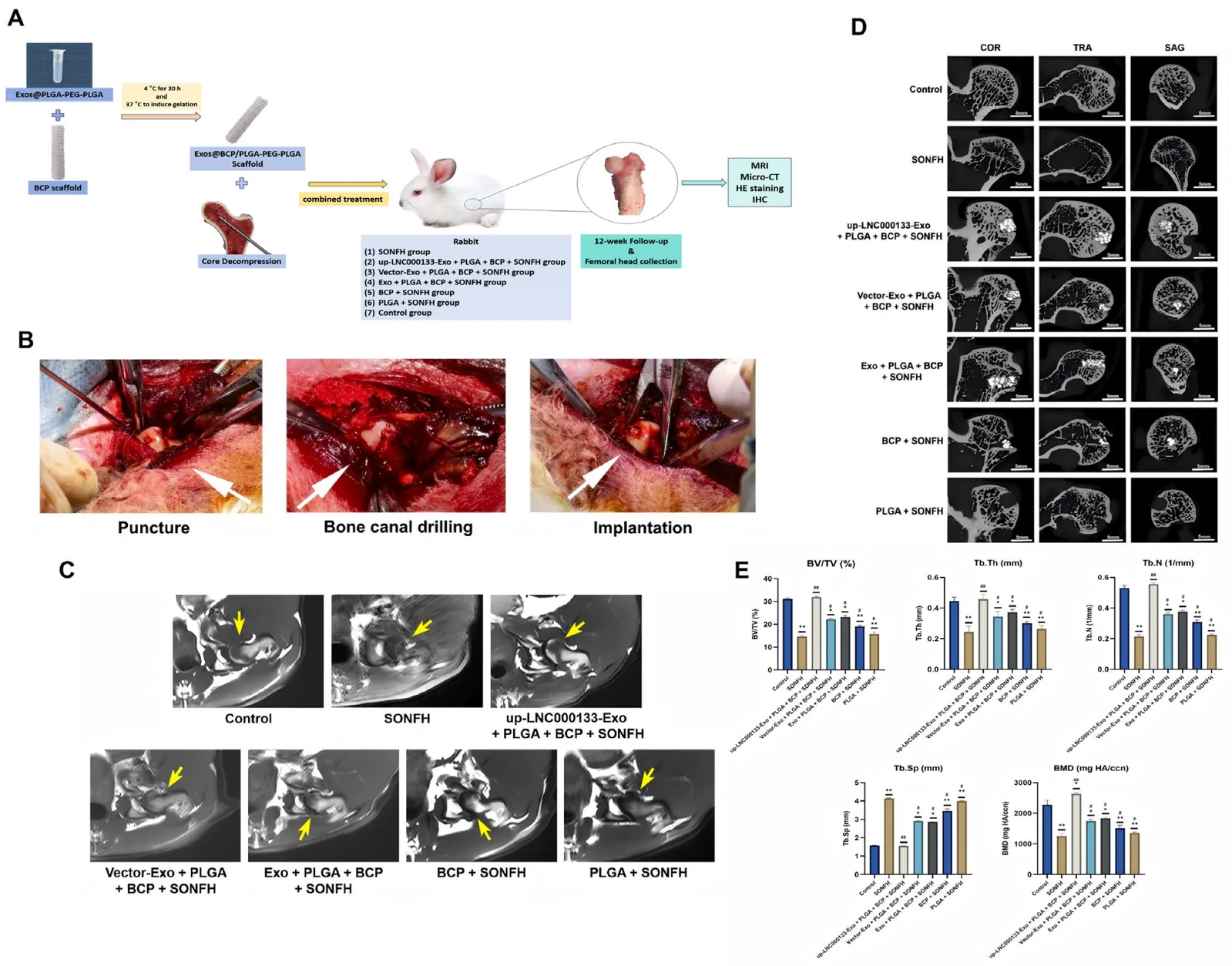
**Imaging evaluation of LNC000133-Exos-loaded BCP/PLGA-PEG-PLGA scaffolds for the treatment of SONFH in rabbits.** (**A**) Schematic illustration of the animal experimental design; (**B**) representative images illustrating the scaffold transplantation procedure; (**C**) representative MRI images showing femoral head signals in rabbits from each group after 12 weeks of treatment; (**D**) representative micro-CT images showing subchondral bone structures of the femoral heads in each group after 12 weeks of treatment (scale bar = 5 mm); (**E**) quantitative analysis of micro-CT parameters. Data are presented as mean ± standard deviation (SD). Note: White arrows indicate the femoral head region in the surgical images, while yellow arrows indicate the femoral head region in the MRI images. * *p* < 0.05 and ** *p* < 0.01 compared with the Control group; ^#^ *p* < 0.05 and ^##^ *p* < 0.01 compared with the SONFH group. MRI, magnetic resonance imaging; LNC000133, long non-coding RNA 000133; PLGA, poly (lactic-co-glycolic acid); BCP, biphasic calcium phosphate; SONFH, steroid-induced osteonecrosis of the femoral head.

To further investigate the therapeutic role of LNC000133-Exos in SONFH, rabbits were treated as described in Section 4.17, followed by MRI and micro-CT imaging analyses, along with HE and IHC staining. The overall experimental design is illustrated in Figure 5A and the surgical procedure is illustrated in Figure 5B.

After 12 weeks of treatment, T2-weighted MRI revealed abnormally high subchondral signals in the femoral heads of rabbits in the SONFH group, indicative of bone marrow edema and osteonecrosis, confirming successful establishment of the SONFH model. Compared with the SONFH group, femoral head edema was alleviated in the Exo+PLGA+BCP+SONFH and Vector-Exo+PLGA+BCP+SONFH groups. Notably, the up-LNC000133-Exo+PLGA+BCP+SONFH group exhibited more pronounced reduction in edema, whereas little or no improvement was observed in the BCP+SONFH and PLGA+SONFH groups (Figure 5C). These findings demonstrated that implantation of LNC000133-Exos-loaded BCP/PLGA-PEG-PLGA scaffolds effectively ameliorated femoral head pathology in SONFH rabbits. Furthermore, this therapeutic effect was superior to that achieved with unmodified hBMSC-Exos-loaded BCP/PLGA-PEG-PLGA scaffolds and was substantially greater than that observed with BCP or PLGA-PEG-PLGA implantation alone.

Furthermore, micro-CT analysis revealed that, compared with the Control group, rabbits in the SONFH group exhibited no obvious radiographic signs of femoral head collapse but showed substantial disruption of trabecular architecture, including trabecular thinning, subchondral cyst formation, and bone resorption (Figure 5D). These structural abnormalities were accompanied by significant decreases in BV/TV, Tb.Th, Tb.N, and trabecular bone mineral density (BMD), together with increased Tb.Sp and porosity, confirming successful establishment of the SONFH model. Compared with the SONFH group, trabecular deterioration in the femoral head was alleviated in the Exo+PLGA+BCP+SONFH and Vector-Exo+PLGA+BCP+SONFH groups. Notably, the up-LNC000133-Exo+PLGA+BCP+SONFH group exhibited the most pronounced improvement in trabecular architecture. Quantitative micro-CT analysis further confirmed these trends (Figure 5E). Importantly, the micro-CT findings were consistent with the MRI results.

### 2.7. HE and IHC Staining

**Figure 6:**
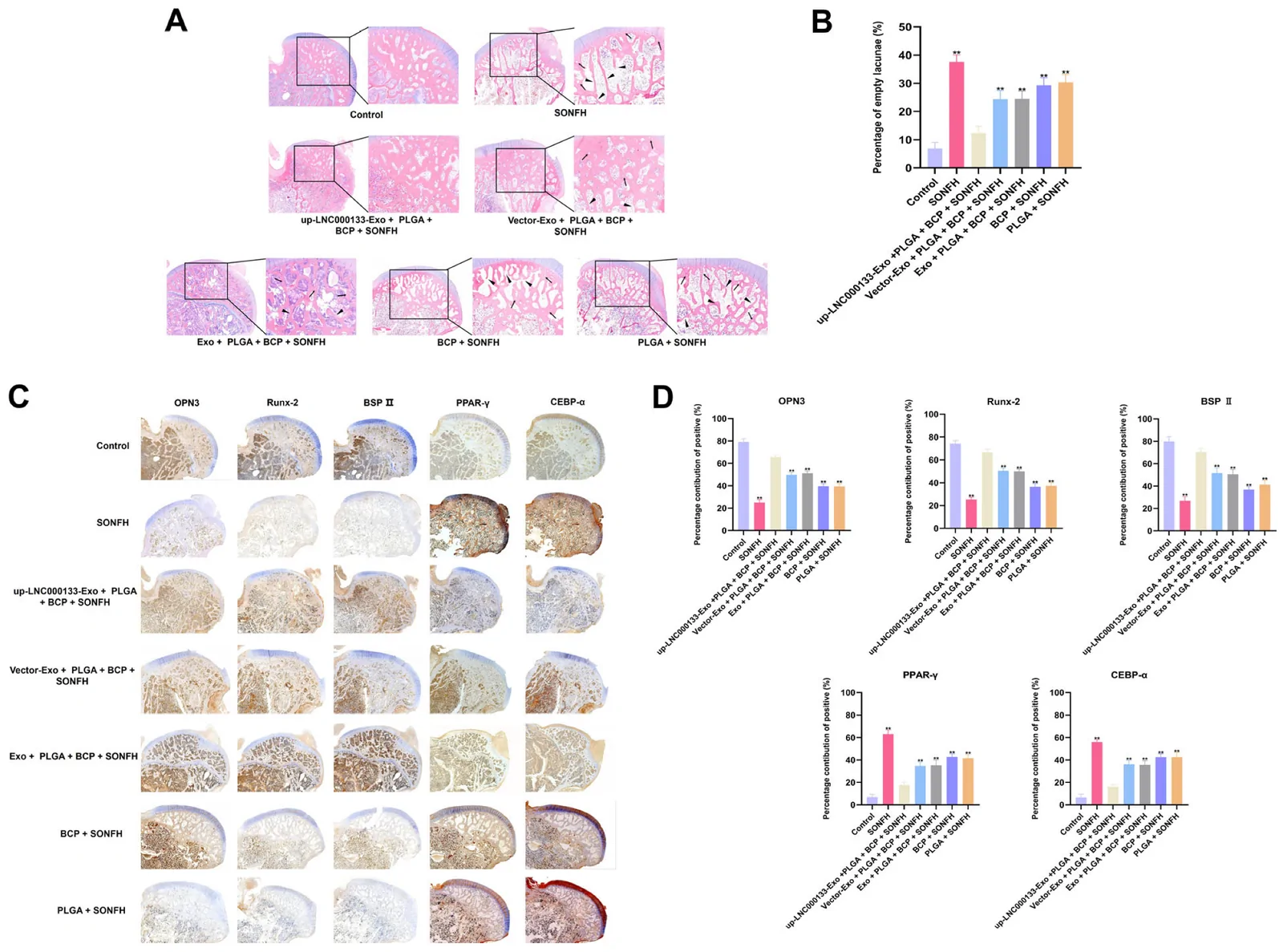
**Histological evaluation of LNC000133-Exos-loaded BCP/PLGA-PEG-PLGA scaffolds in SONFH rabbits.** (**A**) Representative images of HE staining showing subchondral bone structures of the femoral heads in each group after 12 weeks of treatment. (**B**) Quantification of the percentage of empty lacunae in femoral head trabeculae in each group after 12 weeks of treatment. (**C**) Representative IHC images showing the expression of OPN3, Runx-2, BSP II, PPAR-γ, and CEBP-α in the femoral heads of rabbits from each group after 12 weeks of treatment. (**D**) Quantitative analysis of IHC staining. Data are presented as mean ± standard deviation (SD) * *p* < 0.05 and ** *p* < 0.01 compared with the Control group. CEBP-α, CCAAT/enhancer-binding protein-α; HE, hematoxylin and eosin; IHC, immunohistochemistry; LNC000133, long non-coding RNA 000133; PLGA, poly(lactic-co-glycolic acid); BCP, biphasic calcium phosphate; SONFH, steroid-induced osteonecrosis of the femoral head; OPN3, osteopontin 3; Runx-2, runt-related transcription factor 2; BSP II, bone sialoprotein II. Arrows indicate trabecular thinning and loosening; arrowheads indicate nuclear pyknosis and empty lacunae.

HE staining was performed to evaluate histopathological changes in the femoral head. Pronounced osteonecrotic lesions were observed in the SONFH, PLGA+SONFH, and BCP+SONFH groups, characterized by sparse and thinned subchondral trabeculae, extensive empty lacunae containing pyknotic osteocyte nuclei, and marked adipocyte infiltration accompanied by fibrous tissue proliferation. In contrast, these pathological changes were substantially alleviated in the Exo+PLGA+BCP+SONFH and Vector-Exo+PLGA+BCP+SONFH groups, as indicated by fewer necrotic cavities, reduced nuclear pyknosis, and partial restoration of trabecular thickness. The most pronounced histological improvement was observed in the up-LNC000133-Exo+PLGA+BCP+SONFH group, in which trabecular architecture was largely preserved and closely resembled that of normal bone (Figure 6A). Consistently, quantitative analysis revealed that the percentage of empty lacunae was significantly reduced in the up-LNC000133-Exo+PLGA+BCP+SONFH group compared with the SONFH group (Figure 6B). Immunohistochemistry (IHC) staining was subsequently performed to assess the expression of osteogenic markers (OPN3, Runx-2, and BSP II) and adipogenic markers (PPAR-γ and CEBP-α). Compared with the SONFH, PLGA+SONFH, and BCP+SONFH groups, the up-LNC000133-Exo+PLGA+BCP+SONFH group exhibited markedly increased expression of OPN3, Runx-2, and BSP II, whereas the Exo+PLGA+BCP+SONFH and Vector-Exo+PLGA+BCP+SONFH groups showed moderate increases in these markers (Figure 6C). Conversely, expression of the adipogenic markers PPAR-γ and CEBP-α was markedly decreased in the up-LNC000133-Exo+PLGA+BCP+SONFH group, whereas intermediate expression levels were observed in the Exo+PLGA+BCP+SONFH and Vector-Exo+PLGA+BCP+SONFH groups (Figure 6D). Together, these findings demonstrate that implantation of LNC000133-overexpressing hBMSC-derived exosome-loaded BCP/PLGA-PEG-PLGA scaffolds substantially alleviated osteonecrotic damage, enhanced osteogenesis, and suppressed adipogenesis in vivo. Moreover, this therapeutic strategy achieved superior outcomes compared with unmodified hBMSC-derived exosome-loaded BCP/PLGA-PEG-PLGA scaffolds and transplantation of BCP scaffolds or PLGA-PEG-PLGA hydrogels alone.

## 3. Discussion

SONFH remains a major challenge in hip-preserving treatment, largely because impaired osteogenic capacity of BMSCs compromises bone regeneration, accelerates femoral head collapse, and ultimately necessitates THA [1,4]. Despite extensive investigation, definitive therapeutic targets for SONFH have yet to be established, owing to its multifactorial pathogenesis involving vascular dysfunction, dysregulated lipid metabolism, impaired osteogenic differentiation of BMSCs, apoptosis, and genetic polymorphisms [2,19]. With the rapid advancement of nanomedicine, exosome-based therapies have emerged as promising approaches for SONFH due to their ability to deliver bioactive cargos and mediate intercellular communication [6,20,21]. Nevertheless, further optimization is required to enhance their therapeutic efficacy, targeting specificity, and delivery efficiency.

Against this backdrop, our previous studies combined next-generation sequencing with bioinformatics analyses to establish the RNA expression profile associated with SONFH in hBMSCs, identifying numerous differentially expressed genes (DEGs) associated with SONFH, followed by preliminary validation. In vitro experiments further demonstrated that LNC000133-Exos enhanced exosome uptake by osteoblasts and promoted their proliferation and osteogenic differentiation through regulation of the LNC000133/miR-362-5p/TGF-β3/SMAD3/BMP2 signaling axis [9].

Beyond the imbalance between osteogenic and adipogenic differentiation, impaired proliferative capacity and excessive apoptosis of hBMSCs are also critical pathological events contributing to SONFH progression [22]. Prolonged or excessive glucocorticoid exposure markedly inhibits hBMSC proliferation, induces apoptosis, impairs osteogenic differentiation, and promotes adipogenic differentiation. These pathological changes collectively lead to reduced functional osteoblast populations, accompanied by increased adipocyte accumulation, bone marrow adiposity, and elevated intraosseous pressure, ultimately resulting in impaired bone repair, progressive trabecular deterioration, and femoral head collapse [23]. Therefore, an effective therapeutic strategy for SONFH should not only preserve hBMSC viability by enhancing proliferation and suppressing apoptosis, but also restore lineage balance by promoting osteogenesis and inhibiting adipogenesis [24]. Coordinated modulation of these processes may improve the local regenerative microenvironment, facilitate bone regeneration within necrotic lesions, and delay structural deterioration of the femoral head [25]. Consistent with this therapeutic rationale, our results demonstrated that LNC000133-Exos-loaded BCP/PLGA-PEG-PLGA scaffolds significantly enhanced hBMSC proliferation while attenuating apoptosis. Meanwhile, neither the BCP scaffold nor the PLGA-PEG-PLGA hydrogel exhibited detectable cytotoxic effects on hBMSCs, indicating the favorable biocompatibility of these scaffold components. Furthermore, LNC000133-Exos-loaded BCP/PLGA-PEG-PLGA scaffolds markedly promoted osteogenic differentiation and suppressed adipogenic differentiation of hBMSCs. Collectively, these findings suggest that LNC000133-Exos-loaded BCP/PLGA-PEG-PLGA scaffolds simultaneously enhance hBMSC survival and regulate lineage commitment, providing a biological foundation for their potential application in SONFH treatment.

Previous studies have demonstrated that injectable PLGA-based hydrogels, owing to their excellent biocompatibility and thermoresponsive gelation properties, can undergo in situ gelation after administration and improve the local retention and stability of bioactive molecules through encapsulation and sustained release. The resulting three-dimensional microenvironment further supports cell viability and facilitates tissue repair [26,27]. Conversely, owing to their favorable mechanical strength, osteoconductive properties, and interconnected porous architecture, BCP scaffolds provide structural support at bone defect sites, serving as a stable framework for new bone formation while promoting the local retention and sustained activity of therapeutic factors through surface adsorption [28,29,30]. The synergistic integration of these two components combines biological activity with mechanical support, thereby generating a more favorable therapeutic microenvironment for bone regeneration. Based on these advantages, we developed a BCP/PLGA-PEG-PLGA scaffold for the delivery of LNC000133-Exos. In a rabbit SONFH model combined with core decompression, implantation of this scaffold markedly delayed disease progression and promoted structural repair of the femoral head, achieving superior therapeutic efficacy compared with scaffolds loaded with unmodified exosomes and substantially greater effects than BCP scaffolds or PLGA-PEG-PLGA hydrogels alone. Compared with systemic intravenous administration, local delivery minimizes nonspecific exosome distribution and clearance by the reticuloendothelial system, thereby reducing off-target organ exposure and enhancing exosome utilization at the lesion site. Furthermore, compared with direct local administration of exosomes, this composite delivery system provides a supportive microenvironment that prolongs exosome stability and bioactivity [31,32]. In addition, exosomes derived from genetically modified BMSCs enable indirect modulation of osteogenesis-related signaling pathways without direct gene transfer, potentially reducing the safety concerns associated with gene therapy approaches [33]. Collectively, our findings demonstrate the feasibility of the BCP/PLGA-PEG-PLGA scaffold as a targeted delivery platform for SONFH therapy, highlighting its potential for enhancing bone regeneration and facilitating clinical translation.

From a translational perspective, the use of LNC000133-engineered exosomes introduces additional considerations beyond those associated with native exosome-based therapies. Engineering the parental BMSCs to enrich a defined RNA cargo adds upstream processing and quality-control requirements, while differences in cell state, engineering efficiency, exosome production, and isolation procedures may contribute to batch-to-batch variation in particle yield, cargo composition, and functional potency [34,35]. Therefore, future development of this platform will require standardized manufacturing procedures and predefined quality attributes for both the exosome product and its LNC000133 cargo. In addition, the stability of engineered exosomes should be evaluated throughout storage, loading, thermally induced gelation, and subsequent release, as processing and material-related factors may influence membrane integrity, cargo retention, and biological activity [34,36].

The incorporation of engineered exosomes into a 3D-printed BCP/PLGA-PEG-PLGA scaffold also adds complexity to manufacturing and scale-up. Reproducible control of scaffold architecture, exosome dose, cargo content, loading efficiency, release behavior, and biological potency will be important when translating this composite system from laboratory-scale preparation to larger-scale production [35,36]. Moreover, an exosome–biomaterial combination product may require coordinated evaluation of the quality, stability, safety, and manufacturing consistency of both components, together with appropriate regulatory strategies. As a result, scalable manufacturing, standardized potency assays, long-term stability studies, and rigorous quality-control frameworks will be necessary before the clinical feasibility of this engineered exosome–scaffold platform can be established [35,37].

Despite the encouraging findings obtained in this study, several limitations warrant further investigation. First, the current in vitro experiments were primarily performed using hBMSCs, whereas the potential effects of LNC000133-Exos-loaded BCP/PLGA-PEG-PLGA scaffolds on other cell types implicated in SONFH progression, particularly osteoblasts and endothelial cells, remain insufficiently characterized. Future studies incorporating these cellular models may provide a more comprehensive understanding of the biological mechanisms underlying this scaffold system. Second, the therapeutic efficacy of LNC000133-Exos-loaded BCP/PLGA-PEG-PLGA scaffolds was evaluated in a rabbit SONFH model. Although this model provides valuable evidence for preliminary efficacy assessment, further validation in large animal models and well-designed clinical studies is required before clinical translation. Third, several critical translational challenges associated with exosome-based therapies, including optimal dose optimization, long-term safety assessment, and pharmacokinetic characterization, remain to be addressed. Comprehensive evaluation of these aspects will be essential for advancing the clinical application of this therapeutic strategy.

## 4. Materials and Methods

### 4.1. Isolation and Culture of hBMSCs

This study was approved by the Ethics Committee of the First Affiliated Hospital of Kunming Medical University, and written informed consent was obtained from all participants before sample collection. Human bone marrow mesenchymal stem cells (hBMSCs) were isolated from femoral bone marrow aspirates collected intraoperatively from patients with steroid-induced osteonecrosis of the femoral head (SONFH) or femoral neck fractures who underwent total hip arthroplasty (THA). Cell isolation and purification were performed using density gradient centrifugation as previously described [38]. Upon reaching approximately 90% confluence, cells were passaged at a 1:2 ratio. Third-passage cells were used for all subsequent experiments.

### 4.2. Phenotypes and Multilineage Differentiation of hBMSCs

The immunophenotype of hBMSCs was characterized by flow cytometry (ACEA NovoCyte 2060R, ACEA Biosciences, San Diego, CA, USA). Briefly, cells were harvested by trypsinization, resuspended in phosphate-buffered saline (PBS) supplemented with 1% fetal bovine serum (FBS), and adjusted to a concentration of 1 × 10^6^ cells/mL. The cell suspension was incubated with fluorescein isothiocyanate (FITC)-conjugated antibodies against CD34, CD45, CD73, and CD90 (BD Biosciences, Milpitas, CA, USA) at 37 °C for 30 min in the dark. After incubation, cells were washed twice with PBS and analyzed by flow cytometry. Unstained cells were used as negative controls.

The multilineage differentiation potential of third-passage hBMSCs was further evaluated through osteogenic and adipogenic induction using commercially available differentiation media (Fuyuanbio, Shanghai, China) according to the manufacturer’s instructions and established protocols [39]. For osteogenic differentiation, hBMSCs were cultured in osteogenic induction medium, which was refreshed every 2–3 days. After 5 days of induction, alkaline phosphatase (ALP) staining was performed to assess early osteogenic differentiation. After 21 days of induction, calcium mineralization was evaluated by Alizarin Red S (ARS) staining. For adipogenic differentiation, hBMSCs were cultured in adipogenic induction medium for 14 days, with medium replacement every 2–3 days, followed by Oil Red O (ORO) staining to assess intracellular lipid droplet formation.

### 4.3. Solation and Characterization of hBMSC-Derived Exosomes

When hBMSCs reached 80–90% confluence, the culture medium was removed, and cells were washed with PBS before being cultured in serum-free medium (Yocon Biotech Co., Ltd., Beijing, China; Cat# NC0103). Conditioned medium was collected every 24 h and stored at −80 °C until further use. Exosomes were isolated from the culture supernatant by ultracentrifugation as previously described [40].

The morphology of isolated exosomes was examined by transmission electron microscopy (TEM) using a TEM analysis kit (E1610, Weihui Biotechnology, Beijing, China) according to previously reported methods [41]. Images were acquired using a HITACHI HT7800 transmission electron microscope (Hitachi, Tokyo, Japan).

The particle size distribution and concentration of exosomes were determined by nanoparticle tracking analysis (NTA) using a ZetaView PMX 110 system (Particle Metrix, Meerbusch, Germany) according to established protocols [42].

Western blotting was further performed to characterize the exosomes by detecting the exosome-associated markers TSG101 and CD81. Briefly, exosome proteins were extracted using pre-chilled RIPA buffer containing 1% protease inhibitor, and protein concentrations were determined using a BCA assay. Equal amounts of total protein (20 μg per lane) were separated by 10% SDS-PAGE using gels prepared with a rapid gel preparation kit (Epizyme, Shanghai, China) and transferred to PVDF membranes. After blocking, the membranes were incubated overnight at 4 °C with primary antibodies against TSG101 and CD81 (CST, Danvers, MA, USA), followed by HRP-conjugated secondary antibodies (ShareBio, Shanghai, China). Protein signals were visualized using an ECL ultrasensitive detection reagent (Epizyme, Shanghai, China).

### 4.4. Cell Transduction

hBMSCs were transduced with recombinant lentiviral vectors encoding LNC000133 for overexpression (up-LNC000133), shRNA-mediated knockdown (sh-LNC000133), or corresponding negative controls (Vector and sh-Control), according to previously described protocols [43]. Briefly, hBMSCs were incubated with lentiviral particles at the indicated multiplicity of infection (MOI) under standard culture conditions to establish stable gene modulation. Following stable transduction, hBMSC-derived exosomes (hBMSC-Exos) were isolated as described above. Three independent biological replicates were included in each group, and all experiments were performed in triplicate.

### 4.5. Design and Fabrication of the BCP Scaffold

A three-dimensional scaffold model corresponding to the bone tunnel created by core decompression of the rabbit femoral head was designed using SolidWorks 2020 (Dassault Systèmes, Waltham, MA, USA) and fabricated by three-dimensional printing (3D printing; 3 mm in diameter × 10 mm in length). The printing parameters included a nozzle diameter of 0.41 mm, layer thickness of 0.33 mm, filament spacing of 0.8 mm, printing speed of 7–10 mm/s, printing angle of 90°, and air pressure of 0.28–0.35 MPa.

BCP scaffolds were fabricated using a solid-state reaction method. Calcium and phosphate precursors were mixed, calcined, ground, and dispersed to generate a slurry, followed by porogen-assisted molding and sintering. The resulting scaffolds were sterilized and stored for subsequent experiments.

### 4.6. Water Absorption Test

The hydrophilicity of BCP scaffolds was assessed by a water absorption test. Scaffolds were immersed in deionized water at room temperature for 5, 10, 15, 20, and 24 h, gently blotted dry, and weighed until a constant mass was achieved. The water absorption rate was calculated as follows:

Water absorption rate = (m_2_ − m_1_)/m_1_ × 100% (1)

where m_1_ represents the dry mass of the scaffold and m_2_ represents the mass of the scaffold after 24 h of immersion.

### 4.7. Mechanical Property Test

The mechanical properties of BCP scaffolds filled with hydrogels at different mass concentrations were evaluated using a universal testing machine (Instron, Norwood, MA, USA) (*n* = 3). Scaffolds were subjected to vertical compression at a rate of 2 mm/min until failure, and the compressive strength and modulus were calculated from the stress–strain curves. Water-immersed BCP scaffolds were used as the control group for comparison.

### 4.8. Preparation of PLGA-PEG-PLGA Hydrogel and LNC000133-Exos@BCP/PLGA-PEG-PLGA Scaffolds

PLGA-PEG-PLGA hydrogel was prepared by dissolving PLGA-PEG-PLGA in PBS at a ratio of 1:5. Purified LNC000133-Exos were mixed with the hydrogel on ice to generate LNC000133-Exos@PLGA-PEG-PLGA. Sterilized BCP scaffolds were immersed in LNC000133-Exos@PLGA-PEG-PLGA at 4 °C for 30 h and subsequently incubated at 37 °C to induce gelation, resulting in the formation of LNC000133-Exos@BCP/PLGA-PEG-PLGA scaffolds. The scaffold microstructure was characterized by scanning electron microscopy (SEM) at an accelerating voltage of 25 kV and a magnification of 3500×. Before in vivo implantation, the samples were briefly immersed in sterile PBS.

To evaluate the protein release profile of different scaffold formulations, PBS, blank BCP scaffolds, PLGA-PEG-PLGA hydrogel, BCP/PLGA-PEG-PLGA scaffolds, and LNC000133-Exos@BCP/PLGA-PEG-PLGA scaffolds were immersed in PBS (pH 7.4) and incubated at 37 °C. At predetermined time points (days 1, 2, 3, 4, 5, 6, and 7), the release medium was collected and replaced with an equal volume of fresh PBS. The protein concentration in each collected release medium was determined using a BCA Protein Quantification Kit (Yeasen Biotechnology, Shanghai, China) according to the manufacturer’s instructions. The absorbance was measured at 562 nm using a microplate reader, and the protein concentration was calculated based on a standard curve generated with bovine serum albumin (BSA).

### 4.9. Experimental Grouping of hBMSCs

To investigate the effects of LNC000133-Exos-loaded BCP/PLGA-PEG-PLGA scaffolds on hBMSCs, an indirect co-culture system was established using Transwell inserts (0.4 µm pore size, NEST, Wuxi, Jiangsu, China). The experimental groups were defined as follows:

1. Control: Untreated cells without additional treatment.
2. up-LNC000133-Exo+PLGA+BCP: Cells were co-cultured with BCP/PLGA-PEG-PLGA scaffolds loaded with LNC000133-Exos using a Transwell system.
3. Vector-Exo+PLGA+BCP: Cells were co-cultured with BCP/PLGA-PEG-PLGA scaffolds loaded with Vector-Exos using a Transwell system.
4. Exo+PLGA+BCP: Cells were co-cultured with BCP/PLGA-PEG-PLGA scaffolds loaded with normal Exos using a Transwell system.
5. BCP: Cells were co-cultured with BCP scaffolds using a Transwell system.
6. PLGA: Cells were co-cultured with PLGA-PEG-PLGA hydrogel using a Transwell system.
7. OS: Cells were cultured in osteogenic induction medium.
8. OS+up-LNC000133-Exo+PLGA+BCP: Cells were co-cultured with LNC000133-Exos-loaded BCP/PLGA-PEG-PLGA scaffolds using a Transwell system and cultured in osteogenic induction medium.
9. OS+Vector-Exo+PLGA+BCP: Cells were co-cultured with Vector-Exos-loaded BCP/PLGA-PEG-PLGA scaffolds using a Transwell system and cultured in osteogenic induction medium.
10. OS+Exo+PLGA+BCP: Cells were co-cultured with normal Exos-loaded BCP/PLGA-PEG-PLGA scaffolds using a Transwell system and cultured in osteogenic induction medium.
11. OS+BCP: Cells were co-cultured with BCP scaffolds using a Transwell system and cultured in osteogenic induction medium.
12. OS+PLGA: Cells were co-cultured with PLGA-PEG-PLGA hydrogel using a Transwell system and cultured in osteogenic induction medium.
13. AS: Cells were cultured in adipogenic induction medium.
14. AS+up-LNC000133-Exo+PLGA+BCP: Cells were co-cultured with LNC000133-Exos-loaded BCP/PLGA-PEG-PLGA scaffolds using a Transwell system and cultured in adipogenic induction medium.
15. AS+Vector-Exo+PLGA+BCP: Cells were co-cultured with Vector-Exos-loaded BCP/PLGA-PEG-PLGA scaffolds using a Transwell system and cultured in adipogenic induction medium.
16. AS+Exo+PLGA+BCP: Cells were co-cultured with normal Exos-loaded BCP/PLGA-PEG-PLGA scaffolds using a Transwell system and cultured in adipogenic induction medium.
17. AS+BCP: Cells were co-cultured with BCP scaffolds using a Transwell system and cultured in adipogenic induction medium.
18. AS+PLGA: Cells were co-cultured with PLGA-PEG-PLGA hydrogel using a Transwell system and cultured in adipogenic induction medium.

The following experiments were performed: Cell Counting Kit-8 (CCK-8) assay, flow cytometric apoptosis analysis, and TUNEL staining were conducted for groups 1–6; ALP staining, ALP activity assays, and ARS staining were performed for groups 1 and 7–12; and ORO staining was performed for groups 1 and 13–18.

### 4.10. CCK-8 Assay

Cell proliferation was assessed using a CCK-8 (ShoreBio, Shanghai, China) assay. hBMSCs from each experimental group were seeded into 96-well plates at a density of 2 × 10^3^ cells per well and cultured in complete medium. At the indicated time points (days 1, 3, 5, and 7), the culture medium was replaced with 100 μL of fresh complete medium containing 10% (*v*/*v*) CCK-8 reagent. The cells were then incubated at 37 °C in a humidified incubator containing 5% CO_2_ for 1 h in the dark. Subsequently, absorbance at 450 nm was measured using a microplate reader (SM600, Yongchuang, Shanghai, China). All experiments were performed in triplicate.

### 4.11. Flow Cytometric Apoptosis Analysis

Cell apoptosis was assessed by flow cytometry using an Annexin V-FITC/PI Apoptosis Detection Kit (Elabscience, Wuhan, China) according to the manufacturer’s instructions. Briefly, hBMSCs were seeded into 6-well plates at a density of 1 × 10^6^ cells per well and cultured under the specified experimental conditions. After 3 days of treatment, cells were collected, stained with Annexin V-FITC and propidium iodide (PI), and analyzed by flow cytometry to quantify apoptotic cell populations. All experiments were performed in triplicate.

### 4.12. TUNEL Staining

Cell apoptosis was morphologically assessed using a TUNEL assay kit (Solarbio, Beijing, China) according to the manufacturer’s instructions. Briefly, hBMSCs were seeded into 6-well plates at a density of 1 × 10^6^ cells per well and cultured under the specified experimental conditions. After 3 days of treatment, cells were fixed and subjected to TUNEL staining. Fluorescence images were acquired using a fluorescence microscope (Olympus, Hachioji, Tokyo, Japan), and six randomly selected fields were analyzed for each sample. Quantification of TUNEL staining was performed using ImageJ software (version 1.54g, National Institutes of Health, Bethesda, MD, USA). Following background subtraction and threshold-based segmentation, nuclei were identified and quantified using the Analyze Particles function. DAPI-positive nuclei were defined as the total cell population, whereas nuclei exhibiting colocalized TUNEL and DAPI fluorescence were identified as apoptotic cells. The apoptotic index was calculated as the percentage of TUNEL-positive nuclei relative to the total number of DAPI-stained nuclei. All experiments were performed in triplicate.

### 4.13. ALP Staining

ALP staining was performed using a commercial kit (Solarbio, Beijing, China) to evaluate osteogenic differentiation of hBMSCs. Briefly, cells were seeded into 24-well plates and cultured under the specified experimental conditions for 5 days. Subsequently, cells were fixed with 4% paraformaldehyde (200 μL per well) for 15 min and incubated with ALP working solution at 37 °C in the dark for 20 min. Nuclear fast red was then applied for 5 min as a counterstain. ALP activity was visualized using a light microscope, with positive staining appearing blue [44]. Cells were washed three times with PBS before each experimental step. All experiments were performed in triplicate.

### 4.14. ALP Activity Assay

ALP activity in hBMSCs was determined using an ALP Activity Assay Kit (Elabscience, Wuhan, China) according to the manufacturer’s instructions. Briefly, hBMSCs were seeded into 96-well plates and cultured under the specified experimental conditions for 5 days. Following treatment, cells were collected and lysed to obtain cell lysates. The lysates were incubated with the reaction reagents at 37 °C for 15 min, followed by the addition of chromogenic reagent to terminate the reaction and develop color. Absorbance was measured at 520 nm using a microplate reader. ALP activity was calculated according to the manufacturer’s instructions. All experiments were performed in triplicate.

### 4.15. ARS Staining

ARS staining was performed to assess osteogenic differentiation of osteoblasts. As previously described [39], cells were seeded into 24-well plates and treated according to the experimental grouping for 21 days. After treatment, cells were fixed with 200 μL of 4% paraformaldehyde for 20 min, followed by staining with 0.2% ARS solution for 15 min. After three washes with PBS, mineralized nodules were visualized using an inverted phase-contrast microscope (Olympus, Hachioji, Tokyo, Japan). All experiments were performed and analyzed in triplicate.

### 4.16. ORO Staining

ORO staining was performed to assess adipogenic differentiation of hBMSCs using an ORO Staining Kit (Solarbio, Beijing, China) according to the manufacturer’s instructions. Briefly, hBMSCs were seeded into 24-well plates and cultured under the specified experimental conditions. After 14 days of adipogenic induction, cells were fixed with 4% paraformaldehyde for 30 min at room temperature, washed three times with PBS, and stained with freshly prepared ORO working solution for 20 min. Excess dye was removed by rinsing with distilled water, followed by hematoxylin counterstaining for 1 min. Images were acquired using a light microscope (Olympus, Japan), and six randomly selected fields were analyzed for each sample. Quantitative analysis of ORO staining was performed using ImageJ software (National Institutes of Health, Bethesda, MD, USA). Following color thresholding, the ORO-positive area was measured and normalized to the total image area, and results were expressed as the percentage of ORO-positive area. All experiments were performed in triplicate.

### 4.17. Rabbit Model of SONFH and Experimental Grouping

All animal experiments were conducted in a specific pathogen-free (SPF) facility and approved by the Institutional Animal Care and Use Committee in accordance with the Guidelines for the Care and Use of Laboratory Animals. A total of 35 healthy male Japanese White rabbits (3–4 months old; 2.5–3.0 kg) were obtained from Kunming Medical University (quality certificate nos. kmu240189 and kmu240233; production license no. SCXK (Dian) K2020-0004) and acclimatized for 1 week before experimentation. All experimental procedures were approved by the Animal Ethics Committee of Kunming Medical University (KMMU20221560). Rabbits were housed under standard conditions at Yunnan Labreal Biotechnology Co., Ltd. (Kunming, China), with free access to food and water. The SONFH model was established by intramuscular administration of methylprednisolone (MPS, 20 mg/kg/day; Pfizer, New York, NY, USA) three times per week for 3 consecutive weeks, as previously described [45]. Following model establishment, animals were randomly assigned to seven groups (*n* = 5 per group): (1) SONFH group—rabbits received intramuscular injections of MPS (20 mg kg^−1^ day^−1^) three times per week for 3 weeks; (2) up-LNC000133-Exo + PLGA + BCP + SONFH group—following SONFH induction, rabbits underwent femoral intramedullary implantation of BCP/PLGA-PEG-PLGA scaffolds loaded with hBMSC-derived exosomes overexpressing LNC000133 (final exosome concentration, 100 μg/mL); (3) Vector-Exo + PLGA + BCP + SONFH group—following SONFH induction, rabbits underwent femoral intramedullary implantation of BCP/PLGA-PEG-PLGA scaffolds loaded with vector-transfected hBMSC-derived exosomes (final exosome concentration, 100 μg/mL); (4) Exo + PLGA + BCP + SONFH group—following SONFH induction, rabbits underwent femoral intramedullary implantation of BCP/PLGA-PEG-PLGA scaffolds loaded with unmodified hBMSC-derived exosomes (final exosome concentration, 100 μg/mL); (5) BCP + SONFH group—following SONFH induction, rabbits underwent femoral intramedullary implantation of blank BCP scaffolds; (6) PLGA + SONFH group—following SONFH induction, rabbits underwent femoral intramedullary implantation of PLGA-PEG-PLGA hydrogel alone; and (7) Control group—rabbits received intramuscular administration of normal saline. All surgical procedures were performed under anesthesia to minimize animal discomfort. The implanted materials within the medullary cavity were 3 mm in diameter and 10 mm in length. After 12 weeks of treatment, rabbits were euthanized by air embolism under anesthesia, and bilateral femoral heads were collected for MRI, micro-computed tomography (micro-CT), haematoxylin and eosin (H&E) staining, and immunohistochemical (IHC) analyses.

### 4.18. MRI Analysis

MRI was performed using a 16-channel longitudinal coil on an Ingenia Elition X 3.0T MRI system (Philips, Amsterdam, The Netherlands) to evaluate abnormal signal changes in the femoral heads of each group. Turbo spin-echo T2-weighted fat-saturated images in the transverse plane were acquired using the following parameters: repetition time (TR), 2500 ms; echo time (TE), 130 ms; slice thickness, 1.5 mm; interslice distance, 0.20 mm; field of view (FOV), 100 mm; and matrix, 144 × 121 pixels.

### 4.19. Micro-CT Analysis

Femoral heads fixed with 4% paraformaldehyde were analyzed using a μCT100 system (SCANCO Medical, Zurich, Switzerland). Micro-CT scanning was performed using the following parameters: scanning energy intensity, 70 kVp and 200 μA; filter, 0.5 AL; matrix, 1024 × 1024 pixels; field of view (FOV), 15.1 mm; resolution, 14.8 μm; and sample time, 250 ms. Two-dimensional images were automatically reconstructed using the micro-CT system. Quantitative analysis of trabecular bone parameters in the femoral heads, including trabecular thickness (Tb.Th), trabecular separation (Tb.Sp), bone volume fraction (BV/TV), and trabecular number (Tb.N), as well as three-dimensional reconstruction images, were obtained using the SCANCO μCT Evaluation Program V6.6 software.

### 4.20. Histological Analysis

Femoral heads from each group were fixed in 4% paraformaldehyde and decalcified in 10% ethylenediaminetetraacetic acid (EDTA). After decalcification for 4 weeks, specimens were dehydrated and embedded in paraffin. Subsequently, 5 μm-thick paraffin sections were prepared in the coronal plane using a microtome (Leica Biocut, Leica Biosystems Nussloch GmbH, Nussloch, Germany) and stained with H&E to evaluate subchondral bone structure. In addition, selected sections were deparaffinized and subjected to immunohistochemical analysis to assess the expression levels of osteopontin 3 (OPN3), Runx-2, BSP II, PPAR-γ, and CEBP-α.

### 4.21. Statistical Analysis

Statistical analyses were performed using SPSS 19.0 software (IBM, Armonk, NY, USA). Quantitative data are presented as mean ± standard deviation (SD). Comparisons between two groups were performed using independent-samples *t*-tests, whereas differences among multiple groups were assessed using one-way analysis of variance (ANOVA). Homogeneity of variance was evaluated using Levene’s test; when variances were unequal, Tamhane’s T2 post hoc test was applied. A *p* value < 0.05 was considered statistically significant. Graphical representations were generated using GraphPad Prism 10.0 software (GraphPad Software, Boston, MA, USA).

## 5. Conclusions

In summary, this study demonstrated that LNC000133-Exos-loaded BCP/PLGA-PEG-PLGA scaffolds effectively enhanced hBMSC proliferation and osteogenic differentiation while modulating cellular behaviors associated with bone regeneration. Furthermore, implantation of LNC000133-Exos-loaded BCP/PLGA-PEG-PLGA scaffolds ameliorated SONFH progression in a rabbit model, highlighting the potential of this scaffold-based exosome delivery platform as a promising strategy for targeted SONFH therapy.

## Data Availability

The data supporting this study’s findings are available from the corresponding author upon reasonable request.
